# Effects of Polymer Morphology on Humic Acid Adsorption onto Model Microplastics and Nanoplastics

**DOI:** 10.1021/acs.langmuir.6c04610

**Published:** 2026-10-15

**Authors:** Cameron D. Smith, Evan J. Easley, Wenlin Zhang

**Affiliations:** Department of Chemistry, 3728 Dartmouth College, Hanover, New Hampshire 03755, United States

## Abstract

Eco-coronae, formed by natural organic matter, such as humic and fulvic acids, on micro- and nanoplastics (MNPs), can enhance surface oxidation, increase human exposure to contaminants, and affect interactions between MNPs and plasma membranes. To better understand the formation of eco-coronae around MNPs, we investigate the influence of crystallinity, an intrinsic property of most commercial semicrystalline plastics, including polyolefins and polyamides, on the adsorption of a model humic acid (HA) in aqueous conditions using all-atom molecular dynamics. Adsorption was found to be thermodynamically favorable across all polymers and morphologies. Crystalline surfaces promote binding by providing planar hydrophobic interfaces that maximize contact with the aromatic groups of HA, and the presence of polar groups on crystalline MNP surfaces enables additional dipole–dipole interactions. Diffusive interfaces of amorphous polyolefins extend the polymer–HA interaction range and, in turn, yield stronger net binding affinities than those of crystalline surfaces. Reduced segmental dynamics in glassy amorphous polyamides hinder structural reorganization upon HA association, mitigating the hydrophobic contacts that drive adsorption. Overall, our simulations demonstrate the effects of MNP surface morphology on HA adsorption and provide molecular-level insights into the factors controlling eco-corona formation on MNPs.

## Graphical abstract

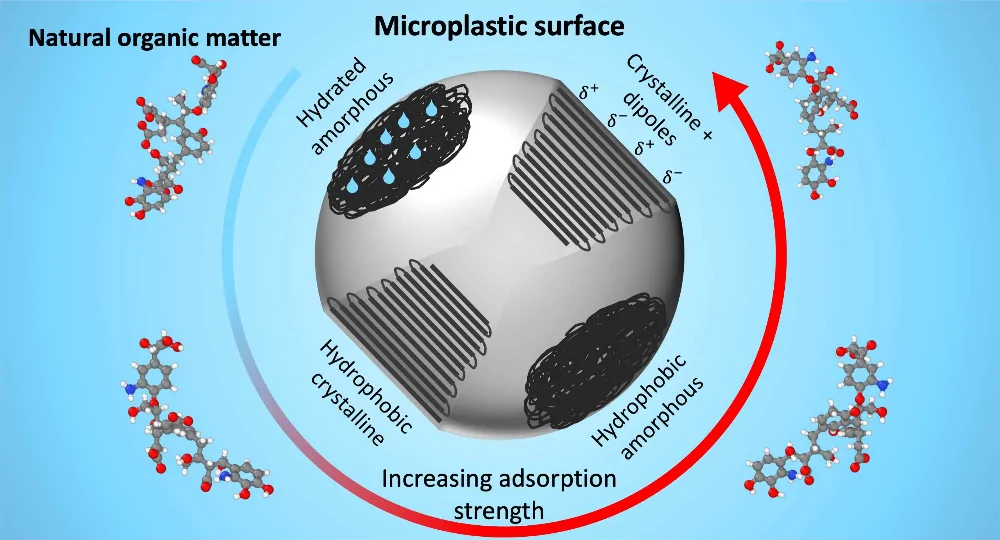

## Introduction

The accumulation and degradation of plastic waste have led to micro- and nanoplastic (MNP) contamination throughout the environment.^1^ Natural organic matter (NOM), formed through microbial decomposition and present in water and soil ecosystems, can adsorb to the surface of MNPs to form an eco-corona.^2–4^ The presence of an eco-corona changes the surface chemistry of the MNPs, accelerates surface oxidation,^5^ and can enhance the formation of biocoronae.^6^ Altering the MNP surface chemistry affects the interaction between the MNP and plasma membranes,^7^ and while pathways of human exposure to these MNPs through ingestion, inhalation, and dermal contact are well-known, the fate of MNPs once in the body is still undergoing considerable investigation.^7–12^ Additionally, the MNPs can act as vectors and increase human exposure to the surface adsorbed species that dissociate when conditions change.^13,14^ Understanding the forces governing eco-corona formation would aid in developing strategies to reduce the risks associated with MNPs.

The accumulation of NOM on the MNP surface is a complex process due to the diversity of NOM substances and heterogeneous polymer compositions. One class of NOM that may contribute to eco-corona formation is the family of humic substances, which are formed through the degradation of natural materials and form a large portion of the NOMs found in the environment.^2,15^ For example, humic acids (HAs) are a subset of humic substances comprised of heterocyclic backbones that are highly soluble in aqueous conditions due to the presence of charged carboxylic acid and oxygen- or nitrogen-containing functional groups.^15^ The molecular architectures of HA enable strong metal binding to clays and minerals, as well as adsorption to polymeric interfaces,^2,13–17^ enabling them to form eco-coronae surrounding MNPs.

Quantifying the interactions between HA and MNP surfaces is essential for understanding the eco-corona formation. Indeed, experimental methods have demonstrated that hydrophobic interactions promote the adsorption of HA onto MNPs, and thus the surface area and morphology of MNPs are key to the adsorption strength.^5,14,16,18^ However, characterizing and correlating the microscopic morphologies of MNP surfaces with HA adsorption is challenging. This is because for common semicrystalline commodity polymers, such as polyethylene, polypropylene, and nylon (polyamide), the surface area, oxidation state, and crystallinity are all convoluted during aging and degradation.

Molecular dynamics (MD) simulations, combined with enhanced sampling methods such as umbrella sampling (US), can provide a molecular level understanding of the assembly and adsorption process in solution.^11,12,19,20^ By sampling along a defined collective variable, such as a center of mass (COM) displacement between a molecule and an interface, US enables the calculation of the potential of mean force (PMF) associated with surface adsorption. This approach facilitates the quantitative characterization of intermolecular interactions and the thermodynamics governing molecular assembly,^20,21^ and has readily been used to study the association between small molecules and a polyethylene interface in aqueous conditions.^22,23^

In this work, we performed all-atom MD simulations to determine the surface adsorption affinity of a model HA on a range of pristine crystalline and amorphous polymer surfaces in aqueous conditions. We find that the adsorption of HA is highly thermodynamically favorable across all polymer types and morphologies, particularly in the presence of both hydrophobic and dipole–dipole interactions. There is a stronger interaction between the HA and the crystalline interface than the amorphous equivalent, which we attribute to the improved contact between the planar surface and the hydrophobic regions in the HA. We further demonstrate that the rubbery nature of the amorphous interface is favorable for HA adsorption, provided water does not permeate the polymer matrix and disrupt the hydrophobic contacts. In contrast, glassy interfaces prevent polymer reorganization, resulting in a weaker attraction by limiting favorable intermolecular contacts. Additionally, steric effects can dramatically influence the adsorption strength. Overall, we identify key factors that impact HA adsorption onto MNPs of commodity semicrystalline polymers. We expect our work here to advance our understanding of eco-corona formation around MNPs.

## Methods

We employed all-atom molecular dynamics (MD) simulations using GROMACS (version 2022.3)^24^ to compute the free energy of association between select polymer interfaces and the Temple–Northeastern–Birmingham (TNB) molecule,^15^ a model HA (Figure 1a and b). Because the highly heterogeneous structures of HAs make them difficult to study directly, the TNB molecule has been proposed as a representative model that approximates the functional groups and chemical composition experimentally observed across a range of HA samples.^15,25^ Therefore, while extrapolating results obtained with TNB to HA in environmental systems is challenging, this simplified representation can provide insight by identifying key interactions that govern the observed behaviors of HA.^13,17^ Carboxylic acid groups on HA samples have a measured p*K*_a_ < 5.^26^ Therefore, we deprotonate the three carboxylic acid groups of the TNB molecule to represent HA at pH ≈ 7.

**Figure 1:**
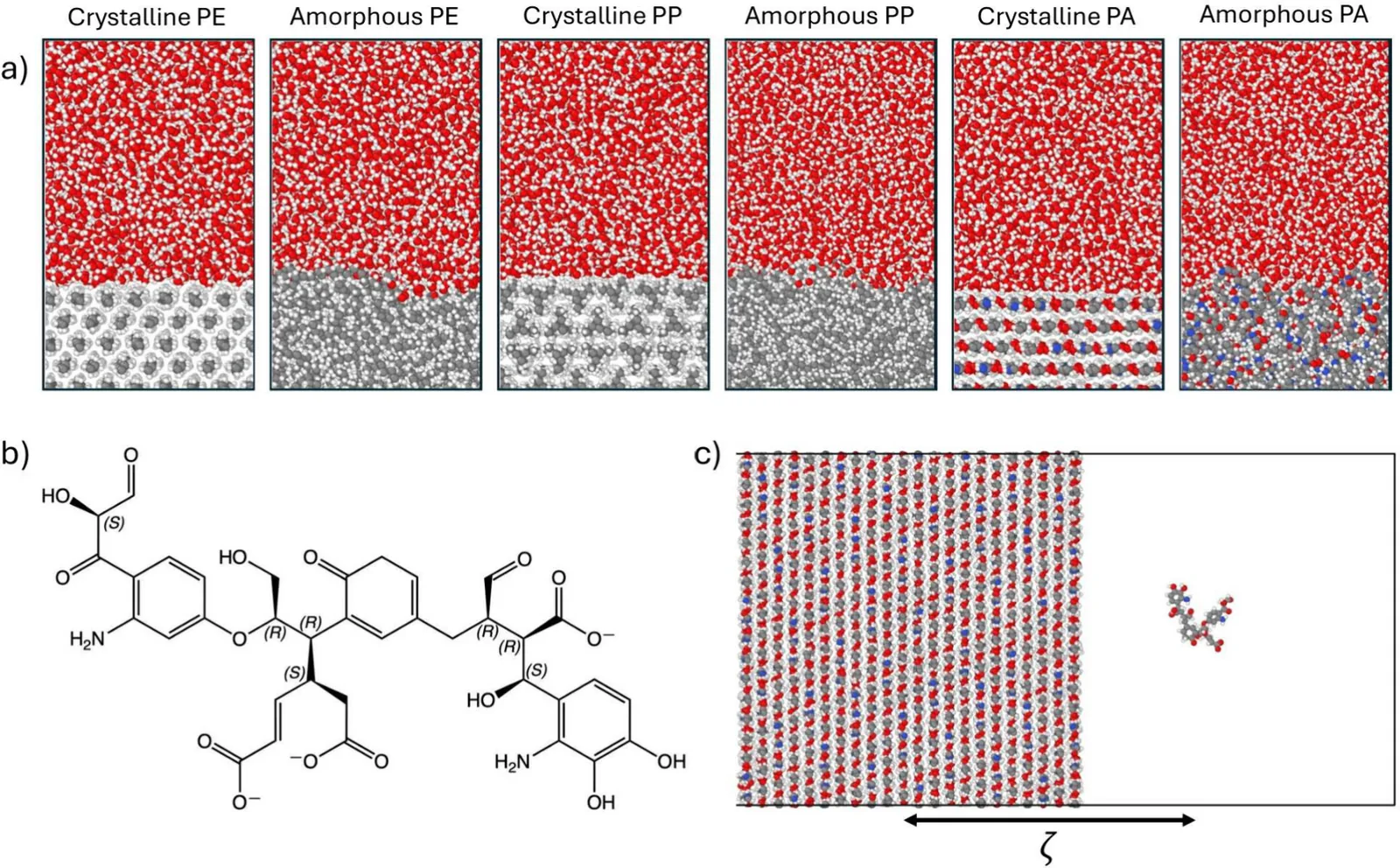
(a) All-atom polymer interfaces, including polyethylene (PE), isotactic polypropylene (PP), and polyamide 6,6 (PA). (b) Molecular structure of the TNB molecule. (c) Collective variable (ζ) as the distance between the center of mass of the interface and TNB molecule (water molecules and sodium counterions removed for clarity). All snapshots are visualized using OVITO^35^ with the color scheme: carbon as gray, hydrogen as white, oxygen as red, and nitrogen as blue.

We utilized periodic boundaries in all directions, and the force fields used were the all-atom CHARMM36 ^27,28^ and the TIP3P water model.^29^ Force field parameters for the polymers were obtained from polymer-builder and the TNB molecule from ligand reader using CHARMM-GUI.^30–32^ A cutoff distance of 1.2 nm was applied to the short-range interactions, with a switching function applied between 1.0 and 1.2 nm for the van der Waals interactions, and the particle mesh Ewald method computed the long-range electrostatic interactions.^33^ With LINCS constraining all bonds to hydrogen,^34^ a time-step of 2 fs was used for all simulations.

### Interface Preparation

We chose a selection of commonly used semicrystalline plastics, polyethylene (PE), isotactic polypropylene (PP), and polyamide 6,6 (PA), to model the polymer–water interfaces that interact with HA. A brief description of the preparation of the amorphous and crystalline interfaces will be used for the sake of brevity. The full description of each system can be found in the Supporting Information.

To prepare the amorphous samples, we created a periodic slab in the *xy* direction with 100 polymer chains and a vacuum region of approximately 13 nm in the *z* direction. The amorphous PE slab was composed of linear low-density PE chains to suppress the formation of crystalline domains,^36^ whereas standard isotactic PP and PA did not crystallize under the preparation conditions. Each free-standing film underwent 40 ns of equilibration above the crystallization temperature using the stochastic velocity rescaling thermostat (v-rescale).^37^ The vacuum regions of the amorphous PE (APE) and PP (APP) samples were solvated and equilibrated for 125 ps in an *NVT* ensemble, followed by another 40 ns *NPT* run at 1 bar using isotropic Berendsen pressure coupling.^38^ The high-temperature equilibration was followed by annealing from 430 to 300 K at −1.0 K ns^–1^ with the v-rescale thermostat and semi-isotropic pressure coupling at 1 bar using the stochastic cell rescale barostat (c-rescale)^39^ to retain the box dimensions in the *z* direction and allow the *xy* plane to fluctuate.

The glassy amorphous PA (APA) was annealed as a free-standing film in vacuum from 540 to 300 K at −2.0 K ns^–1^ with the v-rescale thermostat before being solvated. This was done to prevent mixing between PA and water at the elevated temperature. After annealing, all solvated amorphous films were further equilibrated for 40 ns at 300 K and 1 bar using v-rescale and c-rescale semi-isotropic pressure coupling, respectively.

To create the hydrated APA interface, we took the equilibrated glassy APA interface and ramped the temperature from 300 to 400 K at 2.0 K ns^–1^ to enable water diffusion into the polymer matrix.^40^ The system was held at 400 K for 100 ns before being annealed at −2.0 K ns^–1^ to 300 K. All steps utilized the v-rescale thermostat and isotropic c-rescale pressure coupling at 1 bar. The system was then re-equilibrated at 300 K and 1 bar for 40 ns with the v-rescale and semi-isotropic c-rescale thermostat and barostat, respectively.

Interfaces of crystalline PE (CPE), crystalline PP (CPP), and crystalline PA (CPA) were created to match the experimentally determined crystal structures and approximate thickness of the amorphous films (see the Supporting Information). The polymers were connected across the periodic boundary in the *x* direction to create infinite crystal slabs.^41^ The *z* direction of each box was extended to 23 nm to match the approximate box dimensions of the amorphous interfaces. After solvating the vacuum region of the crystalline slabs, we equilibrated the system by performing *NVT* simulations at 300 K for 1 ns, followed by 1 ns *NPT* runs at 300 K and 1 bar with the v-rescale thermostat and isotropic Berendsen barostat, respectively. Finally, we matched the simulations of amorphous films with further equilibration at 300 K and 1 bar for 40 ns with the v-rescale and semi-isotropic c-rescale thermostat and barostat, respectively.

We use the local orientational order (*S*) of the polymer backbone to identify the presence of any crystalline domains in the amorphous polymer films.^42,43^ The orientation of atom *k*’s neighbors is used to obtain *S*_*k*_ from the local nematic order tensor

1 $$Q_{k}=\frac{3}{2}\left\langle t⊗t\right\rangle -\frac{1}{2}I$$

where ⟨···⟩ denotes the average over all the orientations of the neighboring unit backbone tangent vectors (**t**), ⊗ is the outer product, and **I** is the identity matrix. We use a radial distance cutoff to identify neighbors of *k*. The amorphous PE and PA systems have a similar pairwise radial distribution function [*g*(*r*)] (Figure S2). Therefore, we set the cutoff distance to 1.08 nm to incorporate the second nearest neighbor.^42,43^ We extend the cutoff to 1.32 nm for PP, corresponding to 2*c* where *c* is the crystal lattice parameter in the backbone direction, and use the backbone tangent vector between every three monomers to account for the 3_1_ helices required for PP crystallization.^44^ *S*_*k*_ is the largest eigenvalue of *Q*_*k*_, which can range from 0 (isotropic) to 1 (perfect uniaxial alignment).

### Umbrella Sampling

We placed a TNB molecule at a center of mass (COM) distance of approximately 9 nm from the polymer slab COM in the *z* direction. Three sodium counterions were added to the water to retain charge neutrality. The system was equilibrated for 1 ns at 300 K and 1 bar using v-rescale and semi-isotropic c-rescale thermostat and barostat, respectively. Position restraints of 1000 kJ mol^–1^ nm^–2^ were placed on the heavy atoms of the TNB molecule to prevent diffusion during equilibration. Under the same *NPT* conditions as equilibration, a harmonic potential of 2000 kJ mol^–1^ nm^–2^ was applied to the COM of the TNB molecule and used to pull the molecule toward the slab along the *z* direction at a rate of 0.001 nm ps^–1^. This resulted in the collective variable (ζ) being the COM displacement along the *z* direction. Configurations were extracted every 0.1 nm along ζ to undergo umbrella sampling.

We performed an additional 500 ps equilibration on each configuration before conducting umbrella sampling with the same harmonic potential and *NPT* conditions as the pulling step. Each window greater than 1.2 nm from the interface was simulated for 50 ns, while windows within 1.2 nm underwent 130 ns production runs. The potential of mean force (PMF) along ζ was recovered using the weighted histogram analysis method (WHAM) with a bootstrap analysis creating 200 PMFs to generate the average profile and associated standard deviation for each interface.^45^

## Results and Discussion

To ensure that each crystalline interface retained the correct configuration during setup, we extracted the lattice parameters during the last 20 ns of equilibration at 300 K. All parameters are within 5% of the literature values, indicating good agreement with experimental measurements (Table S2). We also obtained the density profiles of the polymer slabs and water, symmetric about the center of the interface, using the GROMACS density functionality with a bin width of 0.1 nm (see Supporting Information for density profiles). The average density across the polymer interface showed good agreement with the experimental densities of the amorphous and crystalline films (Table 1). The density results, combined with the crystalline lattice parameters, indicate that the CHARMM36 force field provides a suitable representation of the polymers tested.

**Table 1:** Average Polymer Density (ρ) and Interfacial Width for Each Interface^a^

polymer	interface	this work ρ (g cm^–3^)	literature ρ (g cm^–3^)	interfacial width (nm)
polyethylene	amorphous	0.854	0.855	0.46
	crystalline	1.03	1.00	0.01
polypropylene	amorphous	0.854	0.850–0.854	0.42
	crystalline	0.941	0.946	0.01
polyamide 6,6	amorphous	1.07	1.09	0.58
	hydrated amorphous	1.04		0.87
	crystalline	1.25	1.24	0.01

a Literature values for polyethylene, polypropylene, and polyamide 6,6 densities were taken from the *Polymer Handbook*.^46^.

The interfacial width of each system was obtained by fitting the density profiles with the hyperbolic tangent function

$$\rho_{i}\left(z\right)=0.5\left({\mathrm{\rho ̅}}_{i,i}-{\mathrm{\rho ̅}}_{i,j}\right)\left[\tanh\left(-az+c\right)+1\right]+{\mathrm{\rho ̅}}_{i,j}$$ 2

where ρ̅_*i*,*i*_ is the average density of species *i* in the bulk of *i*, ρ̅_*i*,*j*_ is the average density of species *i* in the bulk of species *j*, and *a* and *c* are parameters that fit the interfacial thickness and shift in the *z* direction, respectively. The term ρ̅_*i*,*j*_ is 0 for all interfaces except the hydrated APA system in which water is present in the PA matrix. We can extract the interfacial thickness of each polymer–water interface from the density fits using the 10–90 thickness rule, which states the approximate interfacial width is the distance between ρ_*i*_(*z*) = 0.1 ρ̅_*i*,*i*_ and ρ_*i*_(*z*) = 0.9 ρ̅_*i*,*i*_.^47^ We determined the interfacial widths using both the water and polymer density profiles, and report the average of the two values as the interfacial width in Table 1.

The density fits for all the crystalline slabs effectively behave like step-functions, resulting in interfacial thicknesses of less than the resolution of the density profile (≈0.01 nm). A sharp interface is expected at the crystal-water interface due to the inability of the water to diffuse into the crystalline slab.

The amorphous polyolefin samples exhibit a more diffuse interface than the crystalline samples, yet the interfacial widths remain small at 0.4–0.5 nm for APE and APP. A small interfacial width is expected due to the high immiscibility of water and polyolefins. These values match the width of alkane-water interfaces found in previous experimental and simulation studies.^48,49^

Polyamides, on the other hand, are hygroscopic. Therefore, water can diffuse into the amorphous regions of PA when exposed to increased humidity or directly immersed.^50^ The presence of water in the PA matrix drops the glass transition temperature (*T*_g_) of PA from 55 °C in dry conditions to below 0 °C when the water content reaches 6 wt %, shifting the bulk properties from glassy to a rubbery state at standard temperatures.^51^ We sampled APA in both the glassy state and hydrated states. The hydrated APA sample had an average water content of 4.4 wt % across the entire slab, which approximates a rubbery APA in the broad interfacial region being sampled. The glassy APA interface was slightly more diffuse than the polyolefins at 0.58 nm, while the hydrated APA extended the interfacial width to 0.87 nm.

We used the local orientational order parameter (*S*) to ensure no crystalline regions formed during the annealing and equilibration process. Figure 2 shows a clear delineation in *S* between the amorphous slabs, with *S* < 0.75 for all polymers, and the crystalline interfaces where *S* > 0.95. This indicates that no crystalline domains have formed during annealing and equilibration in the amorphous samples. This is expected with the regular branching in the PE chains inhibiting crystal formation,^36^ and the difficulty in crystallizing PP from melt in simulations.^52^ The rapid quenching of the APA film at −2.0 K ns^–1^ from above the crystallization temperature to below *T*_g_ successfully prevented the formation of any crystalline regions.

**Figure 2:**
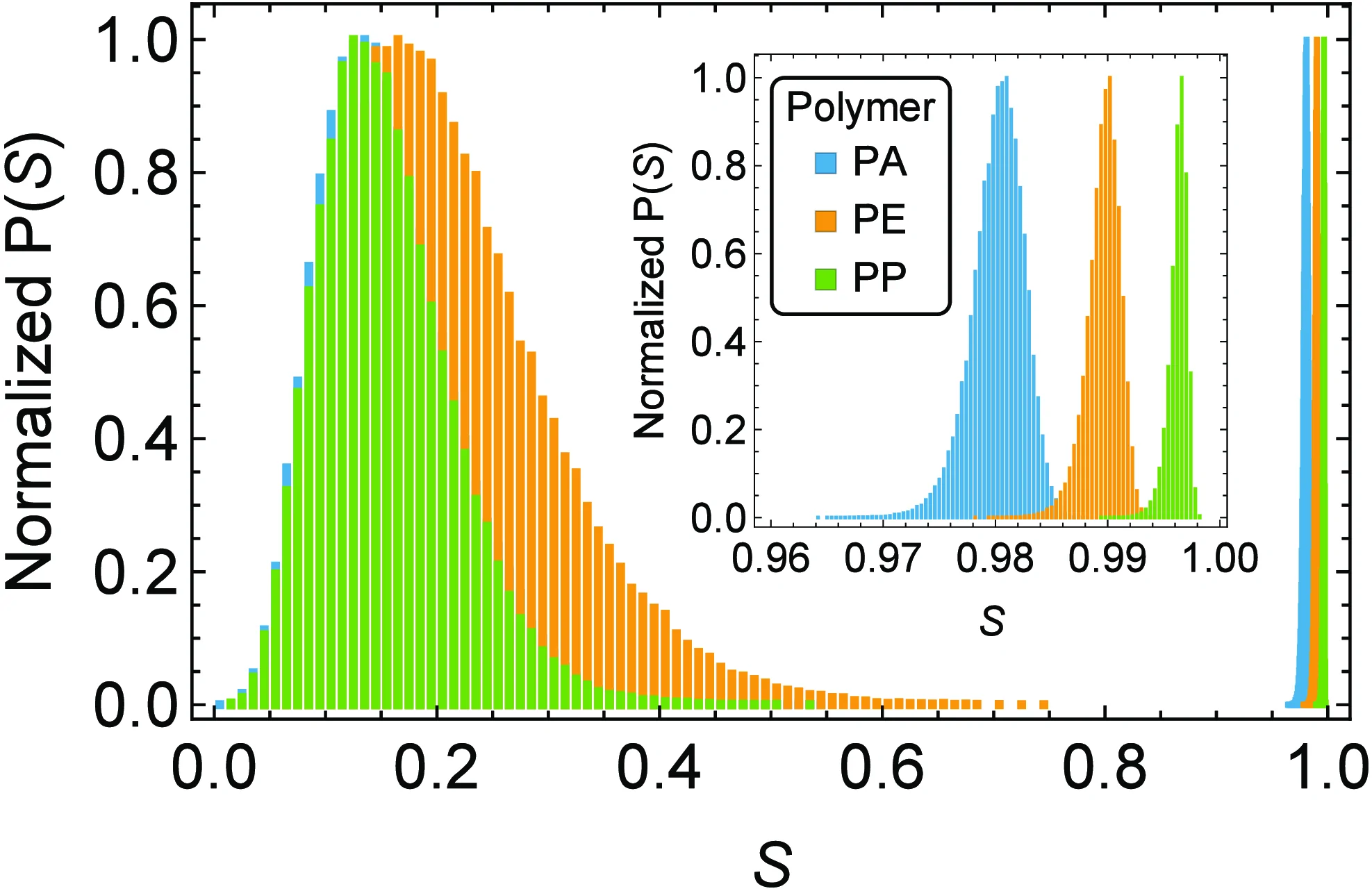
Local orientational order (*S*) of the polymers in each system with clear separation between the *S* of the amorphous polymer films (*S* < 0.75) and crystalline interfaces (*S* > 0.95). The inset magnifies *S* for the crystalline slabs.

After we confirmed that each interface was crystalline or amorphous, we determined the role of crystallinity on the thermodynamics of HA adsorption. We computed the PMF profiles for the association of a TNB molecule with each interface using WHAM (Figure 3). All PMFs show the TNB molecule starts to become attracted to the interface at a separation of around 1.2 nm and that TNB adsorption is favorable, with PMF_min_ ≤ −5 kT for all interfaces.

**Figure 3:**
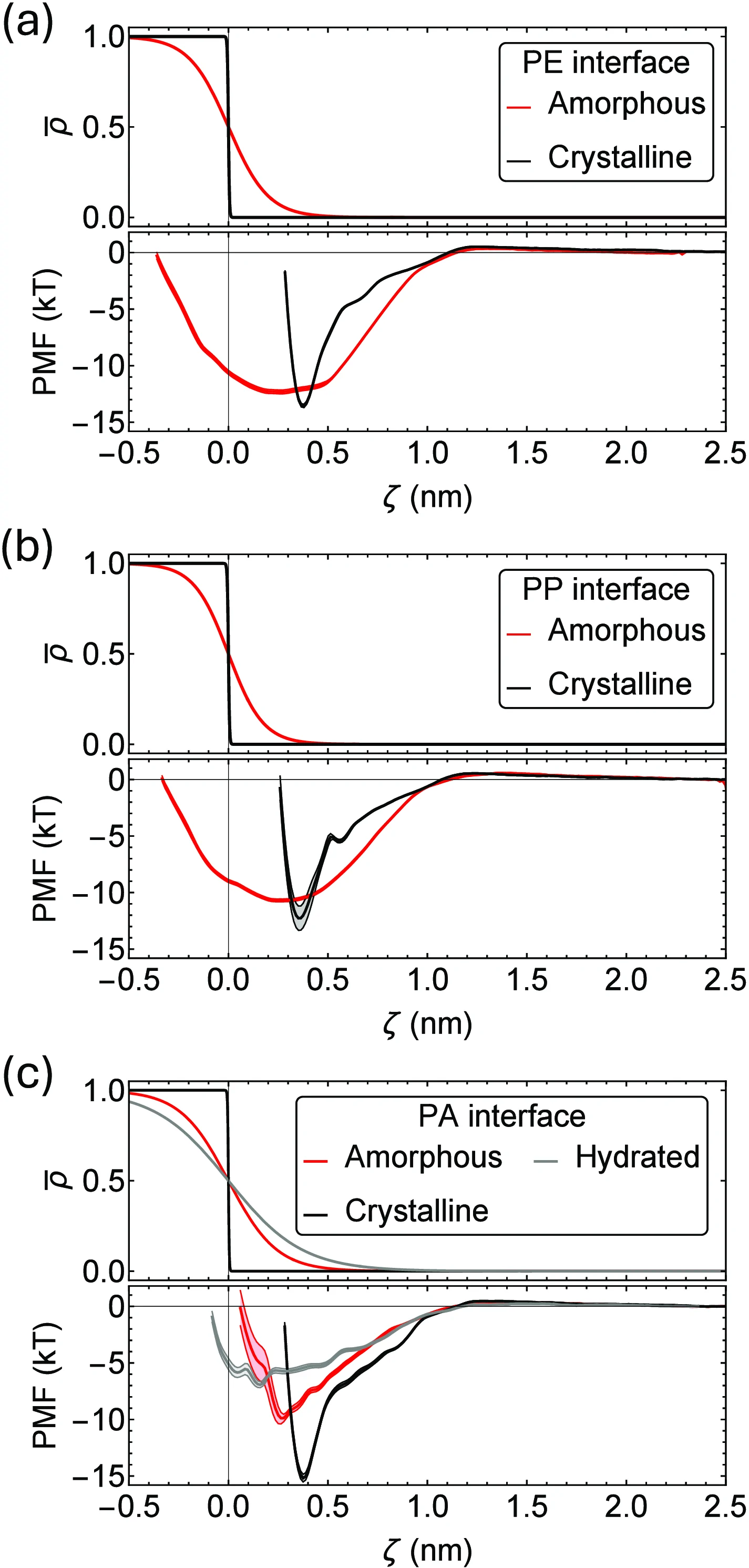
Density and potential of mean force (PMF) profiles for the interfaces tested: (a) PE, (b) PP, and (c) PA. Shading surrounding each PMF represents the standard deviation obtained from bootstrapping.

The APE and APP interfaces produce a broader PMF profile than that of the crystalline samples. This broad profile is due to the increase in the surface area of the diffuse interface, which has been attributed to the increased adsorption capacities of aged microplastics (MPs) compared to virgin samples.^14,16^ The PMF profiles of HA near the APE and APP surfaces show similar shapes and minima, which is expected as both polymers consist of hydrophobic saturated carbon backbones and only differ by the presence of methyl groups on the PP.

The PMF of HA near glassy APA is narrower than the PMFs sampled in APE and APP systems, despite the interfacial width of the APA being larger (Table 1). Upon the incorporation of water into the APA matrix (hydrated PA), the PMF profile broadens, yet the overall adsorption affinity decreases substantially compared with the glassy interface. These findings indicate that the dynamic nature of the hydrated PA enables the interface to reorganize upon HA adsorption, extending the range of attractive interactions. However, the presence of water within the matrix weakens the hydrophobic interactions that drive adsorption and results in an overall reduction in adsorption strength. In contrast, the amorphous polyolefins remain intrinsically hydrophobic and therefore do not require comparable interfacial reorganization to interact favorably with HA. Together, these results highlight a delicate balance between interfacial adaptability, which broadens the range of attractive interactions, and the preservation of hydrophobic character that governs the overall strength of adsorption.

We expected the increase in binding affinity (lower PMF_min_) for the crystalline interface compared to the amorphous interface to arise from an improvement in contact between the rigid crystalline polymer and aromatic rings on the TNB molecule. To identify the primary contacts between the TNB and each interface, we calculated the pairwise distance between each TNB heavy atom and its nearest interfacial heavy atom neighbor throughout the umbrella sampling trajectory at the PMF minimum. A cutoff of 0.5 nm was used to define interfacial contacts, and the probability of each TNB heavy atom being in contact with any polymer heavy atom over the course of the simulation was subsequently determined (Figure 4).

**Figure 4:**
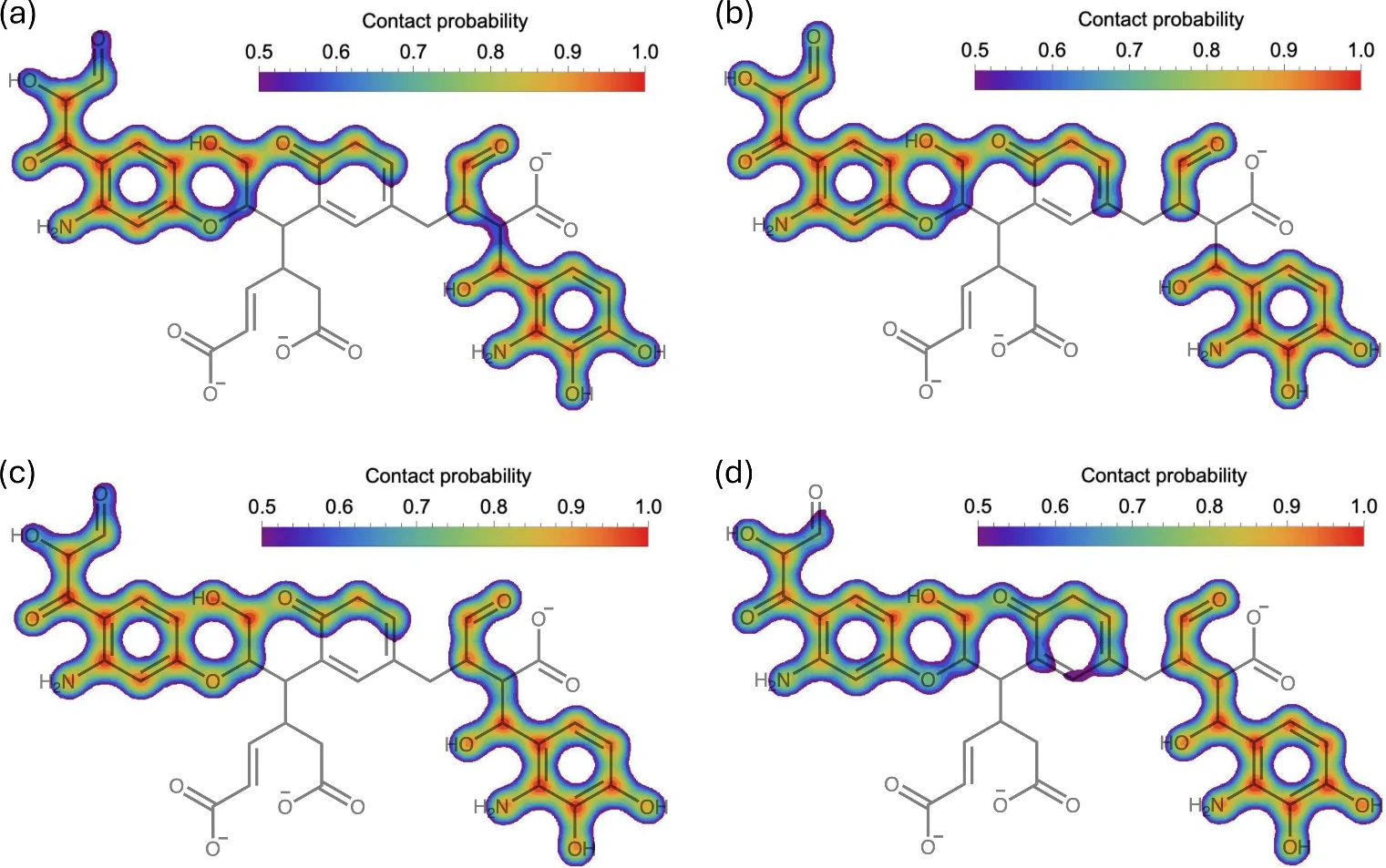
Heatmaps showing the most favored contacts between the TNB molecule and polymer interfaces. (a) Crystalline and (b) amorphous polyethylene and (c) crystalline and (d) glassy amorphous polyamide 6,6. Contact probability is described in the text.

TNB adsorption onto each polymer interface is indeed facilitated through hydrophobic contacts. The heatmaps show that interactions with each interface primarily occur through the aromatic rings, which lie planar against the surface, while the deprotonated carboxylic acid groups remain exposed to the surrounding solvent, as expected.

We observe very little difference between the crystalline and amorphous contact maps (contact maps for PP are shown in Figure S3). This is not surprising for the PE and PP interfaces, which exhibited only a 1.0–1.5 kT difference in PMF_min_ between the amorphous and crystalline morphologies. There is a slight decrease in contact probability between the CPA and APA surfaces, where both aromatic rings show significant interaction with the CPA film (Figure 4c) while only one ring shows perpetual contact for the APA interface (Figure 4d). The reduced hydrophobic contacts decrease the binding affinity of HA onto glassy APA interfaces.

To better demonstrate the role of hydrophobic interactions in TNB adsorption, we compute the change in solvent-accessible surface area (SASA) of TNB and the crystalline polymer slab along the reaction coordinate ζ (Figure 5). Prior work demonstrated that the hydrophobic interaction per change in SASA of hydrophobic molecules upon contact is 24 cal mol^–1^ Å^–2^.^53^ We therefore expect the hydrophobic attraction between TNB and polymer surfaces to scale linearly with the change in SASA (ΔSASA), although the constant of proportionality should be less than 24 cal mol^–1^ Å^–2^ due to the amphiphilic nature of TNB. Nonetheless, we observe that ΔSASA becomes more negative as the TNB molecule approaches the polymer surface, which contributes to the observed effective attraction. The ΔSASA profiles also closely resemble the corresponding PMFs.

**Figure 5:**
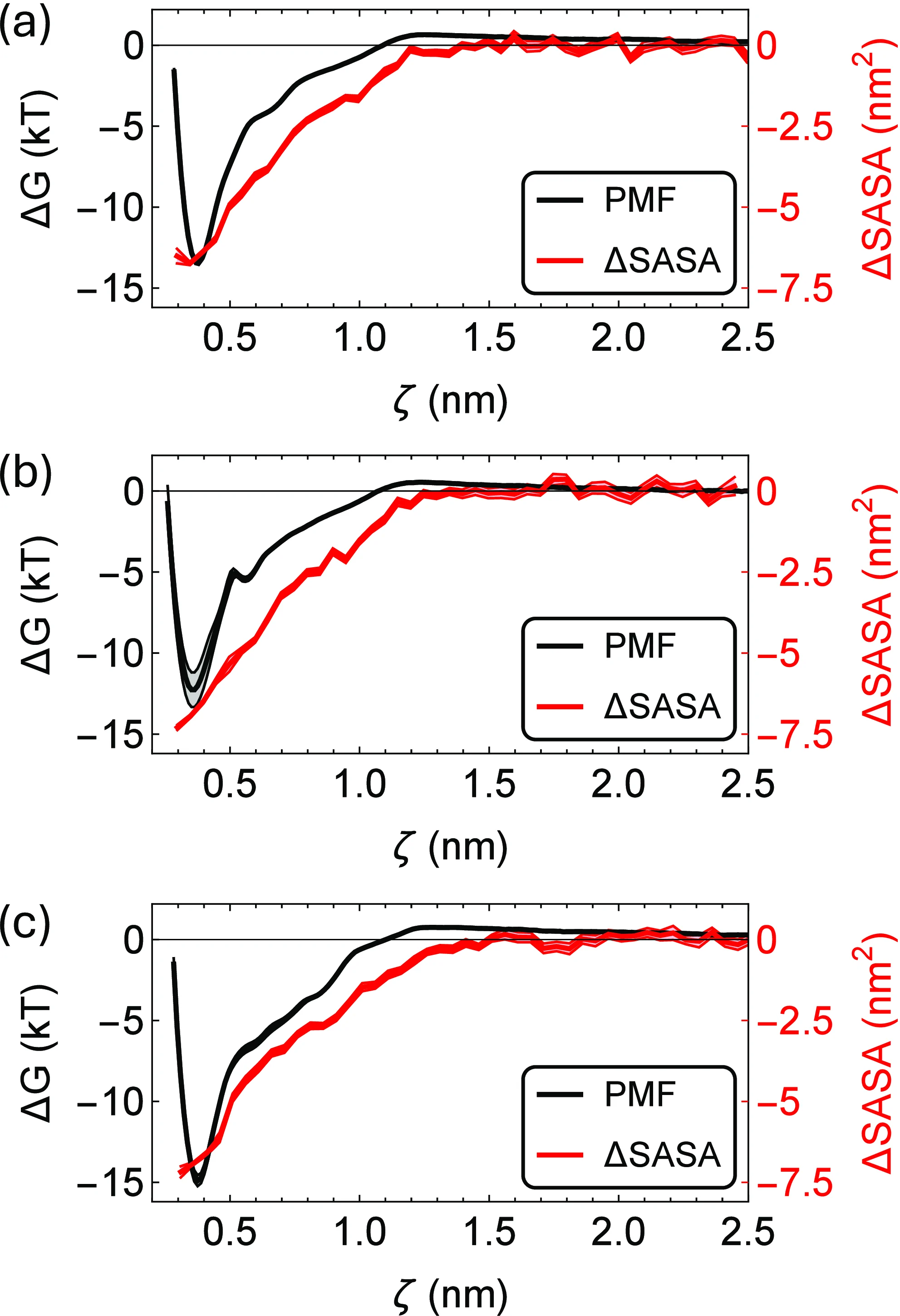
Change in solvent-accessible surface area (SASA) as TNB approaches crystalline surfaces (red curves) of (a) PE, (b) PP, and (c) PA. PMFs sampled using umbrella sampling are shown as references (black).

The PA film differs from the polyolefins due to the presence of polar amide groups along the polymer backbone. These dipoles can facilitate dipole–dipole interactions with the polar groups on the TNB molecule, which explains the stronger affinity for CPA (PMF_min_ ≈ −15 kT) than with either crystalline polyolefin (PMF_min_ ≈ −13 kT) and may contribute to the experimental results which show a higher HA accumulation on PA MPs.^14^

By identifying the species that each TNB atom is paired with, we isolated the propensity to coordinate with the polar amide group (through the amide carbon, nitrogen, or oxygen) or the hydrophobic carbon backbone (Figure 6). We determined the coordination value as the probability of forming a contact with a carbon backbone atom minus the probability of forming a contact with an amide atom [*P*(carbon) – *P*(amide)]. This can range from −1, indicating contact with an amide group throughout the trajectory, to 1, which means pairing with the hydrophobic carbon. A value of 0 can mean no contact throughout, as is the case for the carboxylic acid groups, or an equal contribution from each group.

**Figure 6:**
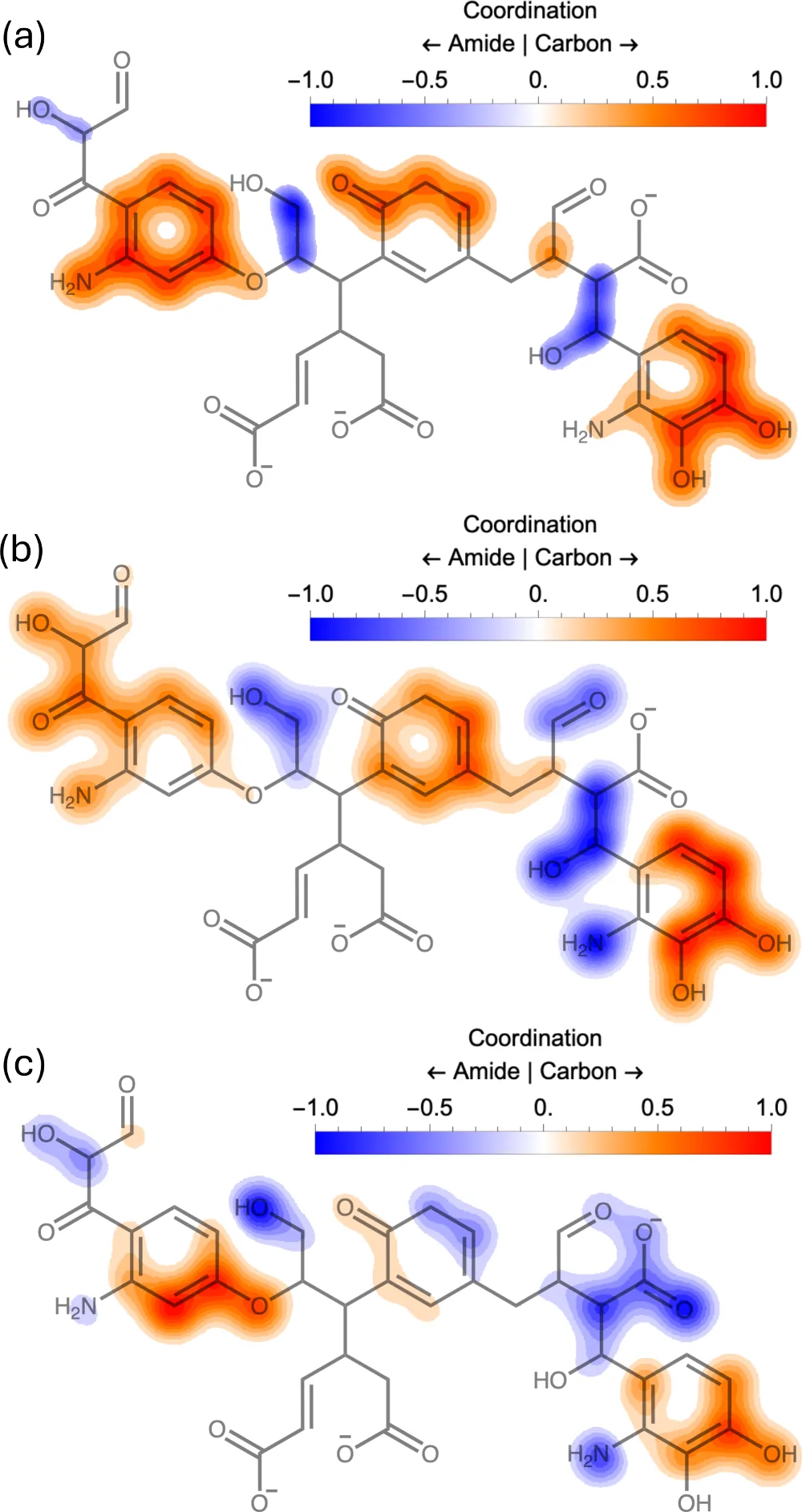
Heatmaps showing the coordination between the TNB molecule and the hydrophilic (amide) and hydrophobic (carbon) regions on the (a) crystalline, (b) glassy amorphous, and (c) hydrated amorphous polyamide 6,6 interfaces.

All ring structures show a strong affinity for pairing with the hydrophobic region on all PA interfaces, with the CPA contact map showing significantly better coordination than the glassy and hydrated APA on the hydrophobic rings. The increase in hydrophobic pairing between the TNB molecule and the CPA is likely driving the stronger attraction observed in the PMF when compared to both APA interfaces. We also see significant polar-group coordination between the aldehyde, amine, and hydroxyl groups on the TNB with the amide in all PA morphologies, which accounts for the stronger binding with CPA than CPE or CPP.

Introducing water into the PA matrix reduces coordination between the hydrophobic regions of the TNB molecule and the APA. The dramatic influence of the presence of water in the PA polymer matrix explains the increase in PMF_min_. As the hydrated PA interfaces become less hydrophobic, TNB adsorption leads to a smaller reduction of the effective SASA upon adsorption, about half that observed for the adsorption onto hydrophobic surfaces. The reduced SASA change thus weakens the binding strength, as observed in the PMFs (Figure 3c).

The results so far concern the most stable TNB conformer (SRRSRRS).^15,54^ However, the presence of seven stereocenters in the TNB molecule enables us to investigate the influence of molecular chirality on surface adsorption. To this end, we generated a second TNB conformer (SSSRSSS) and compared its adsorption behavior with that of the more stable conformer at each polymer–water interface. This comparison enables us to assess the effect of chirality on the adsorption strength of TNB at the different interfaces.

There is a significant preference for the adsorption of the more stable TNB conformer (SRRSRRS) with a PMF_min_ almost twice as strong as that of the other conformation on CPE (Figure 7a). Interestingly, every interface tested also showed a strong preference for the more stable conformation (Figure S4). We produced contact heatmaps for the CPE trajectories to elucidate the origin of the dramatic difference in adsorption strength (Figure 7b and c). It was obvious that the more stable conformer was able to optimize the favorable contacts between the aromatic rings and the interface, whereas sterics prevented the second TNB molecule from forming the same level of coordination and resulted in weaker binding to the surface, reinforcing the hydrophobic driving force of surface adsorption. Additionally, it is critically important for researchers using computational methods to identify the chirality of the molecule used when simulating TNB, as it can have significant effects on the results.

**Figure 7:**
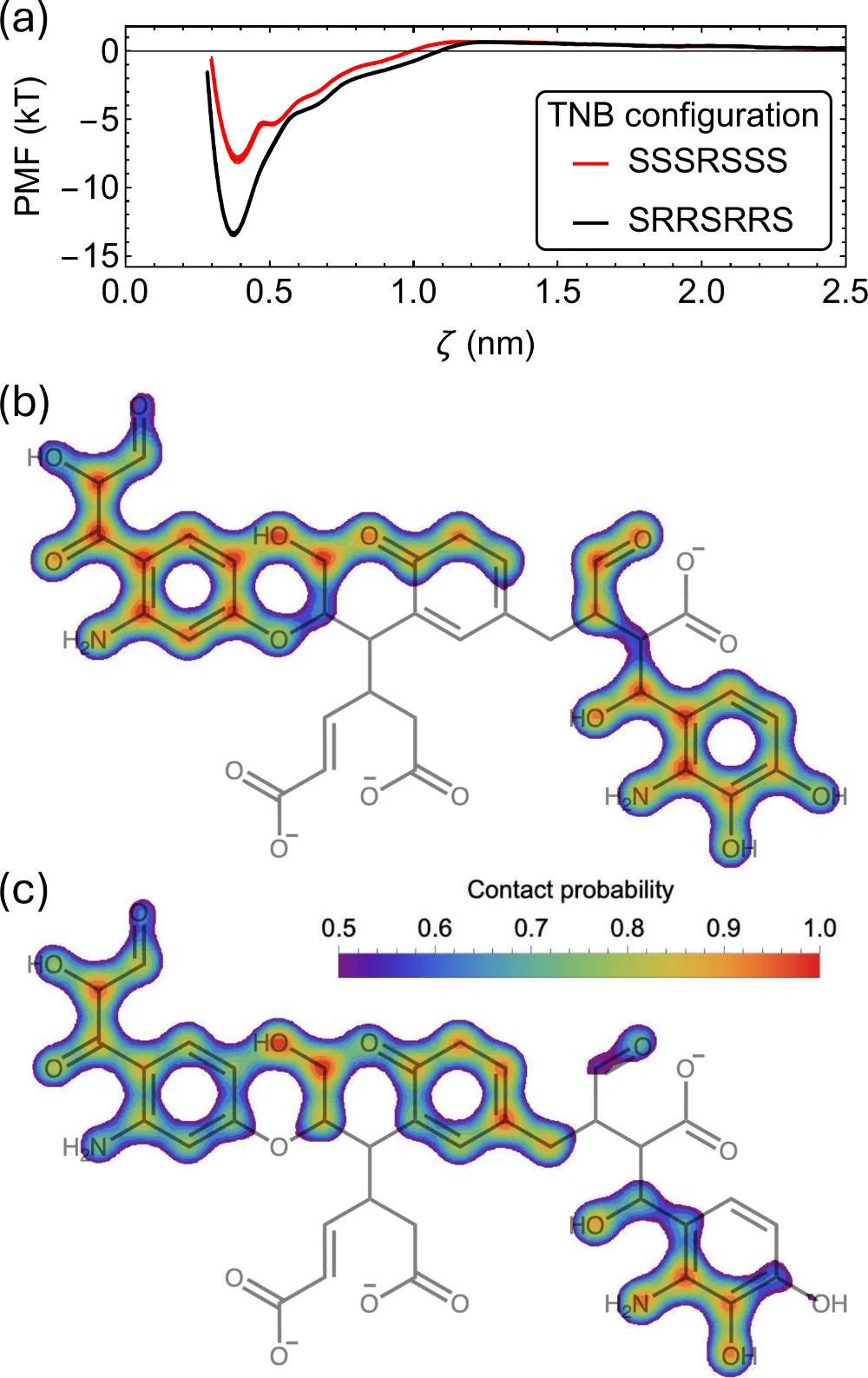
Influence of chirality on adsorption strength and contact with a crystalline PE interface. (a) PMF profiles of TNB molecules with different chiralities. Heatmap showing the most favored contacts for the (b) SRRSRRS and (c) SSSRSSS conformers of the TNB molecule.

We computed the adsorption equilibrium constant (*K*_eq_) for each interface from the PMFs using the method derived by Comer et al.^22,55^

3 $$K_{\mathrm{eq}}=S_{A}\int e^{-\mathrm{PMF}\left(\zeta \right)/kT}d\zeta$$

where *S*_A_ is the surface area and PMF­(ζ) is the free energy profile. We integrate over the range of the attractive basin for each PMF profile. This method has successfully recovered the experimentally measured *K*_eq_ values for small molecules adsorbing onto graphene surfaces and PE interfaces.^22,55^

The interfacial surface area (*S*_A_) was estimated using the alpha-shape surface mesh functionality in OVITO, with a probe radius taken from the first peak of the *g*(*r*) plot.^35,56^ The largest increase in *S*_A_ was observed for the hydrated APA interface relative to CPA, corresponding to an approximately 12% increase due to surface roughness. Given the small change in *S*_A_ values between the amorphous and crystalline samples, and the minimal effect they have on the determination of *K*_eq_, we assume *S*_A_ is equivalent across all interfaces. Additionally, no bias potential was applied parallel to the interface during umbrella sampling. Therefore, the resulting PMF inherently accounts for interfacial roughness. Although the calculated equilibrium constants (*K*_eq_) are not expected to reproduce experimental values, as TNB serves only as a representative model for HA, they provide a consistent basis for comparing the effects of interface morphology and composition. Thus, we determine the relative equilibrium constants *K*_eq_^rel^ = *K*_eq_^*i*^/*K*_eq_^ref^, where *i* is each interface and the PMF of the CPA interface with the SRRSRRS TNB configuration was used as the reference (*K*_eq_^ref^). The corresponding relative free energies of adsorption were then obtained from Δ*G*_rel_ = −ln­[*K*_eq_^rel^] to compare adsorption strength, where a higher Δ*G*_rel_ corresponds to weaker binding.

Figure 8 shows stronger adsorption of the more stable TNB conformer (SRRSRRS) on all interfaces. This is due to the improved hydrophobic contacts between the TNB and the interface. The overlapping uncertainty of Δ*G*_rel_ for the APP and CPP interfaces in Figure 8a obscures any meaningful conclusions on the strength of adsorption. However, adsorption is stronger on APE for both TNB enantiomers, as well as APP for the less stable conformer. This matches experimental data that suggests the higher amorphous content of aged MPs leads to increased HA adsorption affinity on PE and PP.^14,16^

**Figure 8:**
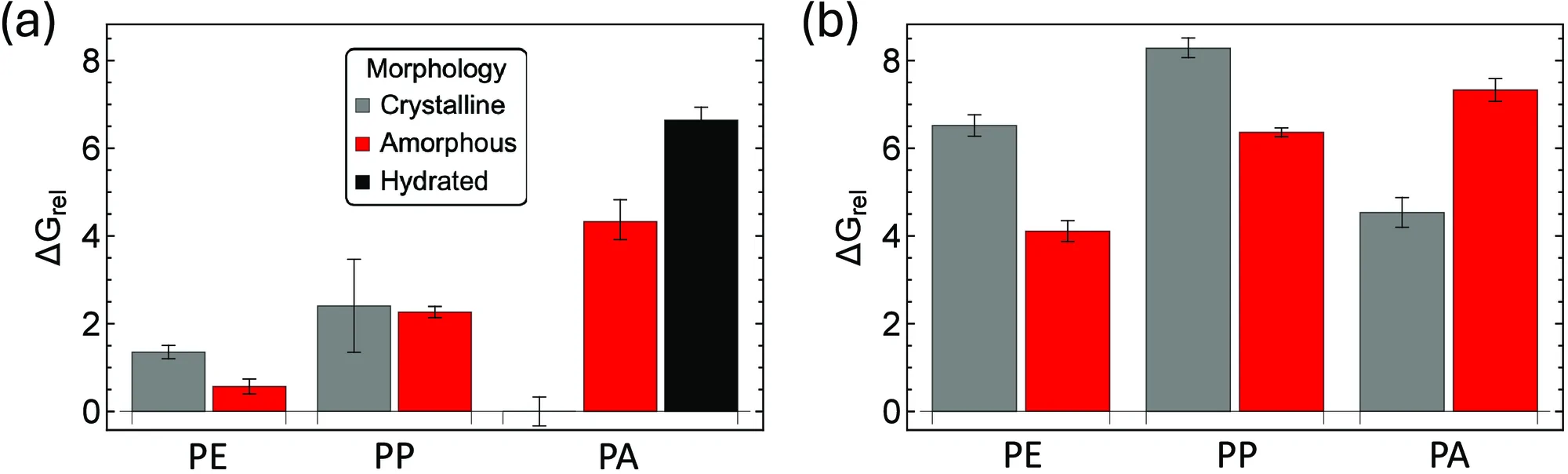
Relative free energy of adsorption (in *kT*) for the (a) SRRSRRS and (b) SSSRSSS TNB conformer.

We observe the opposite trend for the PA interface, with APA having a significantly higher Δ*G*_rel_ for both TNB conformations compared to CPA, and the hydrated APA a higher Δ*G*_rel_ still. The crystallinity of PA has been shown to increase during the aging process,^57^ likely due to higher degradation in the amorphous regions, which leads to the formation of nanoplastics with increased crystallinity.^58^ Therefore, we speculate that the aging-induced increase in crystallinity in NPs could contribute to enhanced HA adsorption on weathered PA. Of course, chain scissions and oxidation can introduce functional moieties to amorphous interfaces, in turn, impacting HA binding strength. Quantifying the effects of different functional groups on HA adsorption is warranted in our future work.

While this study does not account for the potential effects of chain scission or surface functionalization, both of which have been proposed to influence HA adsorption, our results demonstrate that the crystallinity and water content of the MNP alone can substantially affect the strength of HA adsorption. These findings establish that the polymer morphology is a key determinant of adsorption behavior, independent of additional chemical modifications.

## Conclusion

We have performed all-atom MD simulations to elucidate the factors affecting surface adsorption of HA on a range of pristine crystalline and amorphous polymers in solution. We find that the adsorption of HA is highly thermodynamically favorable across all polymer types and morphologies. The sharp interface of a crystalline region improves contact between the planar hydrophobic surface and the aryl groups in HA, and in turn facilitates stronger binding. The amide functionality along the surface of polyamide provides an opportunity for the polar groups on the HA to engage in dipole–dipole interactions in addition to the hydrophobic driving force and results in the strongest interaction tested. Surface oxidation observed on MNPs would also introduce polar functional groups and could enhance HA binding, as previous results suggest.

We also establish that the hydrophobicity of the amorphous interface is important for HA adsorption. Hydrophobic polyolefins in the diffusive interfaces exhibit better contact with the hydrophobic moieties on the TNB molecules, which results in a dramatically broadened attractive region of the PMF profile. Conversely, the glassy amorphous polyamide interface hinders the reorganization of hydrophobic segments upon HA adsorption. The reduced hydrophobic contacts lead to a weaker attractive well in the PMF. Although the presence of water in the polyamide matrix broadens the polymer–water interface and enhances the polymer rearrangement, the hydrated polyamide surface still exhibits a weaker driving force for HA adsorption due to the reduced hydrophobic interactions.

Finally, we show that the chirality of the TNB molecule can impact adsorption strength. Because the TNB molecules approximate more complex natural HA, we expect that stereochemistry may also affect the binding strength of these NOMs to MNPs. Altogether, our findings demonstrate that the adsorption of HA is governed by the intricate interplay between hydrophobic interactions, MNP surface morphology, and molecular chirality. By providing molecular-level insights into HA adsorption at the polymer–water interface, our simulations advance the fundamental understanding of these processes and inspire the development of theoretical models for eco-coronae formation and eco-corona-induced interactions between MNPs and plasma membranes.

## Supplementary Material



## Notes

Details involving the setup of each polymer interface, crystal lattice parameters, pairwise radial distribution functions, interfacial density fits, and TNB chirality PMF profiles (PDF)
