# Supplementary material for: Effects of Polymer Morphology on Humic Acid Adsorption onto Model Microplastics and Nanoplastics
Source: Langmuir. 2026 Oct 15;42(42):31372–81. doi: 10.1021/acs.langmuir.6c04610 (PMC13718613; doi:10.1021/acs.langmuir.6c04610)
Supplement: Supplementary file 1 [file la6c04610_si_001.pdf]

Supplementary Information: Effects of polymer morphology on humic acid adsorption onto microplastics and nanoplastics

Cameron D. Smith, Evan J. Easley, and Wenlin Zhang*

Department of Chemistry, Dartmouth College, Hanover, New Hampshire 03755, USA

E-mail: wenlin.zhang@dartmouth.edu

Contents

1 Setup of polymer interfaces S3

2 Crystal lattice parameters S5

3 Interface density fits S6

4 Pairwise radial distribution functions S7

5 TNB contact maps S8

6 TNB chirality S9

1 Setup of polymer interfaces

The sequence and number of chains used to create each interface can be found in Table S1.

Table S1: Chain sequence and total number of chains used for each interface.

Polymer	Interface	Sequence	# Chains
Polyethylene	Crystalline	$[CH_2]_{72}$	360
	Amorphous	$H[(CH_2)_8(CH_2CH(CH_2CH_3))(CH_2)_8]_{10}H$	100
Polypropylene	Crystalline	$[CH_2CH(CH_3)]_{42}$	208
	Amorphous	$H[CH_2CH(CH_3)]_{66}H$	100
Polyamide 6,6	Crystalline	$[NH(CH_2)_6NHC{=}O(CH_2)_4C{=}O]_5$	396
	Amorphous	$H[NH(CH_2)_6NHC{=}O(CH_2)_4C{=}O]_{15}OH$	100

Amorphous polymer interfaces were formed with linear low-density polyethylene chains (APE), isotactic polypropylene chains (APP), and polyamide 6,6 chains (APA). All amorphous interfaces were periodic in the xy-directions and had a vacuum region of approximately 13 nm in the z-direction. Freestanding polyolefin amorphous thin films were initially equilibrated in a $9.5 \times 9.5 \times 23$ nm box under NVT conditions at 460 K with the stochastic velocity rescale thermostat (v-rescale)[1] for 40 ns.

We solvated the vacuum region of each simulation box and equilibrated each solvated system with NVT simulations at 460 K for 125 ps, followed by 40 ns NPT runs at 430 K and 1 bar with the v-rescale thermostat and isotropic Berendsen barostat,[2] respectively. We then annealed each system from 430 K to 300 K at a cooling rate of -1.0 K ns^{-1} with v-scale thermostat and semi-isotropic stochastic cell rescale barostat (c-rescale)[3] to allow the x/y plane to dissipate stress while retaining the box dimension in the z-direction. The annealed APE and APP slabs were then further equilibrated at 300 K and 1 bar for 40 ns with the v-rescale and semi-isotropic c-rescale thermostat and barostat, respectively. This resulted in slabs with the approximate dimensions of $8.7 \times 8.7 \times 7.2$ nm for APE and $8.6 \times 8.6 \times 7.3$ nm for APP.

The glassy APA thin film was equilibrated for 40 ns in a $8.5 \times 8.5 \times 23$ nm box under NVT

conditions at 540 K to exceed the crystallization temperature of nylon.[4] We annealed the system from 540 K to 300 K at a cooling rate of -2.0 K ns^{-1} with v-rescale thermostat, then solvated the vacuum region of the simulation box to create the interface. The nylon sample was prepared in the vacuum state to prevent mixing with water at the elevated temperature. We then performed a 2 ns NPT run at 300 K and 1 bar with the v-rescale thermostat and anisotropic Berendsen barostat, respectively, followed by a further 38 ns of equilibration at 300 K utilizing semi-isotropic pressure coupling at 1 bar with c-rescale. This resulted an APA slab with the approximate dimensions of $8.4 \times 8.4 \times 7.4$ nm.

To create the hydrated APA interface, we took the equilibrated glassy APA interface and ramped the temperature from 300 K to 400 K at 2.0 K ns^{-1}. A temperature of 400 K was chosen to enable fast water diffusion into the polymer matrix.[5] The system was held at 400 K for 100 ns before being annealed at -2.0 K ns^{-1} to 300 K. All steps utilized the v-rescale thermostat and isotropic c-rescale pressure coupling at 1 bar. The system was then re-equilibrated at 300 K and 1 bar for 40 ns with the v-rescale and semi-isotropic c-rescale thermostat and barostat, respectively. This aging process resulted in a water content of 4.4 wt% in the polymer matrix and slab dimensions of approximately $8.4 \times 8.4 \times 7.6$ nm.

To represent the crystalline polymer regions, we created a periodic slab of crystalline PE (CPE) matching the orthorhombic crystal structure (P_{na2_1} space group).[6] The CPE slab was composed of 20 layers, each consisting of 18 PE chains of 36 monomers, with each polymer connected to itself across the periodic boundary in the x-direction. This resulted in a CPE slab with the approximate dimensions of $9.2 \times 8.6 \times 7.3$ nm.

We formed a periodic crystalline isotactic polypropylene (CPP) slab matching the ideal α_2-form ($P2_1/c$ space group).[7,8] The CPP slab was composed of 16 layers, each consisting of 13 PP chains of 42 monomers, connected across the periodic boundary in the x-direction. This resulted in a CPP slab with the approximate dimensions of $9.2 \times 8.4 \times 8.2$ nm.

Finally, we constructed a periodic crystalline polyamide 6,6 (CPA) slab matching the α-form ($P\bar{1}$ space group).[9] The CPA slab was composed of 22 layers, each consisting of 18

PA chains of 5 repeat units, connected across the periodic boundary in the x-direction. This resulted in a CPA slab with the approximate dimensions of $8.7 \times 8.4 \times 8.0$ nm.

The z-direction of each box was extended to 23 nm to match the approximate box dimensions of the amorphous interfaces. After solvating the vacuum region of the crystalline slabs, we equilibrated the system by performing NVT simulations at 300 K for 1 ns, followed by 1 ns NPT runs at 300 K and 1 bar with the v-rescale thermostat and isotropic Berendsen barostat, respectively. Finally, we matched the amorphous treatment with further equilibration at 300 K and 1 bar for 40 ns with the v-rescale and semi-isotropic c-rescale thermostat and barostat, respectively.

2 Crystal lattice parameters

We extracted the lattice parameters for each crystal slab during the last 20 ns of equilibration at 300 K (Table S2).

Table S2: Lattice parameters for each crystalline slab. Literature values for polyethylene, polypropylene, and polyamide 6,6 taken from *Polymer Handbook*.[10]

Polymer	Lattice parameter	This work	Literature
Polyethylene	a	7.41 ± 0.26 Å	7.42 Å
	b	4.79 ± 0.23 Å	4.95 Å
	c	2.56 ± 0.06 Å	2.55 Å
Polypropylene	a	6.54 ± 0.26 Å	6.66 Å
	b	20.7 ± 0.3 Å	20.8 Å
	c	6.58 ± 0.12 Å	6.50 Å
	β	98.9 ± 1.3 °	99.6 °
Polyamide 6,6	a	4.77 ± 0.12 Å	4.9 Å
	b	5.33 ± 0.21 Å	5.4 Å
	c	17.4 ± 0.1 Å	17.2 Å
	α	48.9 ± 2.1 °	48.5 °
	β	78.3 ± 1.7 °	77.0 °
	γ	66.3 ± 3.5 °	63.5 °

3 Interface density fits

The density profiles shown in Figure S1 were fit using:

$$\rho_i(z) = 0.5(\bar{\rho}_{i,i} - \bar{\rho}_{i,j})\,[tanh(-az + c) + 1] + \bar{\rho}_{i,j} \tag{S1}$$

where $\bar{\rho}_{i,i}$ is the average density of the of species i in the bulk of i, $\bar{\rho}_{i,j}$ is the average density of the of species i in the bulk of species j, and a and c are parameters that fit the interfacial thickness and shift in the z-direction, respectively.

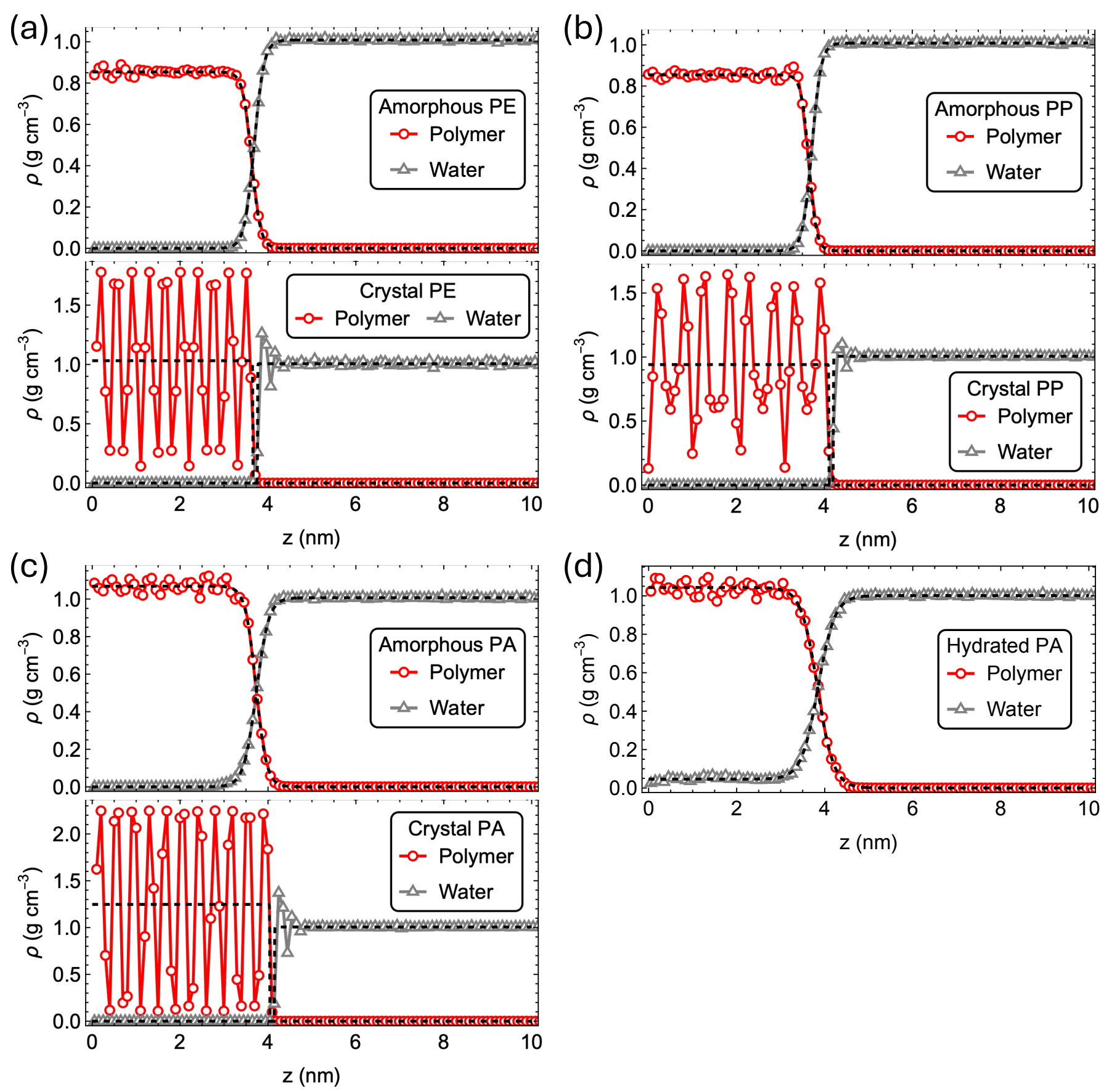

Figure S1: Density profiles and fits for the amorphous (top) and crystalline (bottom) interfaces for (a) polyethylene, (b) polypropylene, (c) polyamide 6,6, and (d) aged amorphous polyamide 6,6.

The extracted densities and interfacial widths are stated in the main text. The rapidly fluctuating density of the crystalline slabs is due to the highly localized mass of the polymers

whose backbones are orientated perpendicular to the z-direction. This backbone orientation results in bins located between neighboring polymers that contain in minimal mass, and bins containing polymers. We performed a moving average with neighboring density points for the CPA and CPE data to aid in visualization, however, the averaging and fit were performed on the original data. The average density over the crystalline domain matches experimental densities for all interfaces.

4 Pairwise radial distribution functions

The pairwise radial distribution functions (g(r)) for the backbone atoms for each amorphous interface are shown in Figure S2.

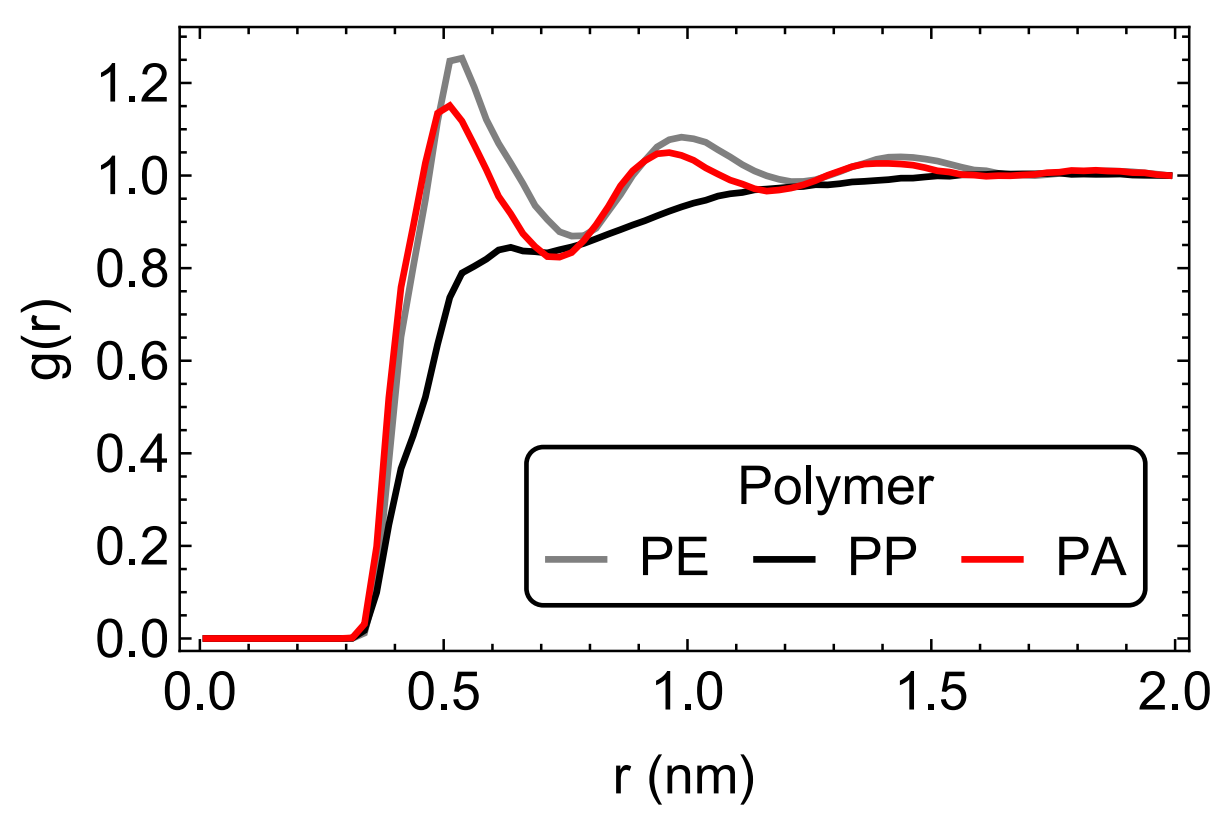

Figure S2: Pairwise radial distribution functions (g(r))

5 TNB contact maps

Contact maps for each polymer interface and the SRRSRRS TNB conformer (Fig. S3).

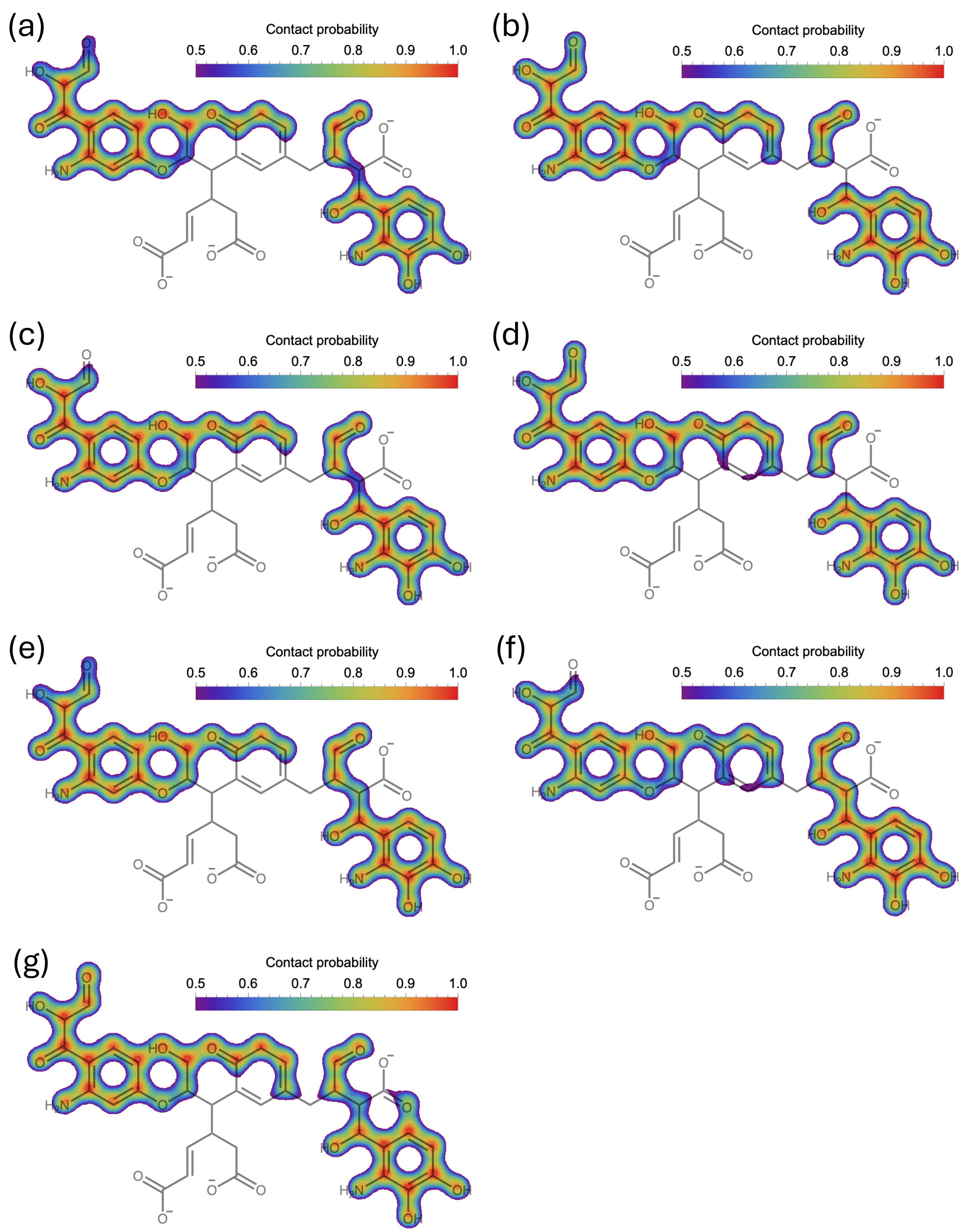

Figure S3: Heatmaps showing the most favored contacts between the TNB molecule and polymer interfaces. (a) Crystalline and (b) amorphous polyethylene, (c) crystalline and (d) amorphous polypropylene, and (e) crystalline, (f) glassy amorphous, and (g) hydrated amorphous polyamide 6,6.

6 TNB chirality

A comparison between the adsorption PMF profiles for two different TNB conformers (Fig. S4).

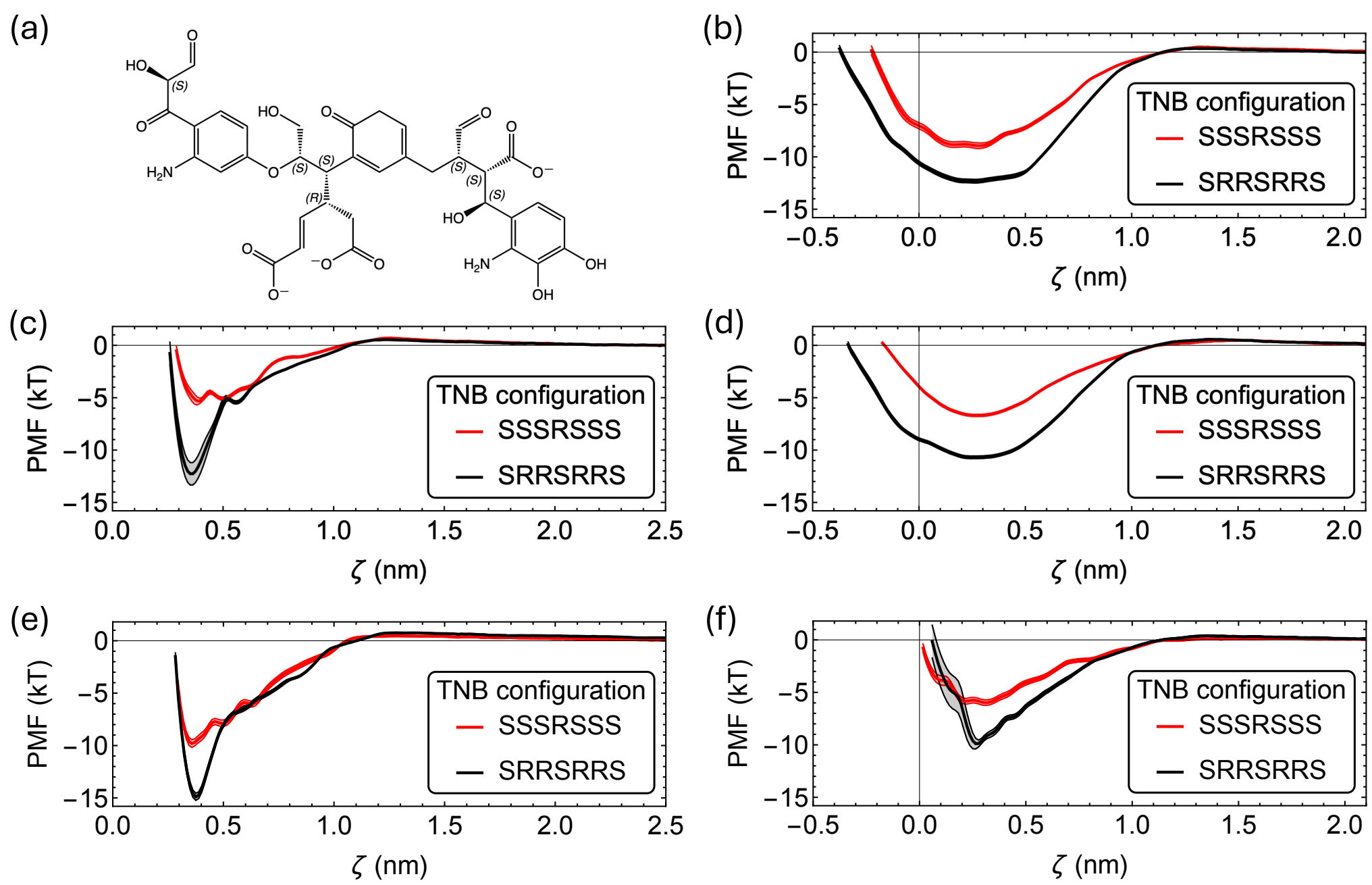

Figure S4: Effect of chirality on TNB adsorption. (a) Assigned chiral centers of the TNB molecule. PMF profiles of the two conformers for (b) APE, (c) CPP, (d) APP, (e) CPA, and (f) APA interfaces.

References

(1) Bussi, G.; Donadio, D.; Parrinello, M. Canonical sampling through velocity rescaling. *The Journal of Chemical Physics* **2007**, *126*, 014101, DOI: 10.1063/1.2408420.

(2) Berendsen, H. J. C.; Postma, J. P. M.; van Gunsteren, W. F.; DiNola, A.; Haak, J. R. Molecular dynamics with coupling to an external bath. *The Journal of Chemical Physics* **1984**, *81*, 3684–3690, DOI: 10.1063/1.448118.

(3) Bernetti, M.; Bussi, G. Pressure control using stochastic cell rescaling. *Journal of Chemical Physics* **2020**, *153*, 114107, DOI: 10.1063/5.0020514.

(4) Ramesh, C.; Kellert, A.; Eltink, S. J. E. A. Studies on the crystallization and melting of nylon 66: 3. Melting behaviour of negative spherulites by calorimetry. *Polymer* **1994**, *35*, 5300–5308, DOI: 10.1016/0032-3861(94)90483-9.

(5) Goudeau, S.; Chariot, M.; Müller-Plathe, F. Mobility enhancement in amorphous polyamide 6,6 induced by water sorption: A molecular dynamics simulation study. *Journal of Physical Chemistry B* **2004**, *108*, 18779–18788, DOI: 10.1021/jp046461e.

(6) Kavesh, S.; Schultz, J. M. Lamellar and interlamellar structure in melt-crystallized polyethylene. I. Degree of crystallinity, atomic positions, particle size, and lattice disorder of the first and second kinds. *Journal of Polymer Science Part A-2: Polymer Physics* **1970**, *8*, 243–276, DOI: 10.1002/pol.1970.160080205.

(7) Mencik, Z. Crystal structure of isotactic polypropylene. *Journal of Macromolecular Science, Part B* **1972**, *6*, 101–115, DOI: 10.1080/00222347208224792.

(8) Auriemma, F.; De Rosa, C.; Malafronte, A.; Scoti, M.; Di Girolamo, R. In *Polypropylene handbook: Morphology, blends and composites*; Karger-Kocsis, J., Bárány, T., Eds.; Springer International Publishing: Cham, 2019; Chapter 2, pp 37–119, DOI: 10.1007/978-3-030-12903-3_2.

(9) Bunn, C. W.; Garner, E. V.; Bragg, W. L. The crystal structures of two polyamides ('nylons'). *Proceedings of the Royal Society A* **1947**, *189*, 39–68, DOI: 10.1098/rspa.1947.0028.

(10) Brandrup, J., Immergut, E. H., Grulke, E. A., Abe, A., Bloch, D. R., Eds. *Polymer Handbook*, 4th ed.; John Wiley & Sons: New York, 1999.
